# Optimization of seismic performance in waste fibre reinforced concrete by TOPSIS method

**DOI:** 10.1038/s41598-023-35495-9

**Published:** 2023-05-21

**Authors:** Husnain Ali, Hafsa Jamshaid, Rajesh Mishra, Vijay Chandan, Petr Jirku, Viktor Kolar, Miroslav Muller, Shabnam Nazari, Khan Shahzada

**Affiliations:** 1 National Textile University, Faisalabad, Pakistan; 2 Department of Material Science and Manufacturing Technology, Faculty of Engineering, Czech University of Life Sciences Prague, Kamycka 129, 165 00 Prague, Czech Republic; 3 Department of Sustainable Technologies, Faculty of Tropical Agriscience, Czech University of Life Sciences Prague, Kamycka 129, 165 00 Prague, Czech Republic; 4 Civil Engineering, University of Engineering and Technology, Peshawar, Pakistan

**Keywords:** Civil engineering, Composites

## Abstract

For a sustainable environment and to tackle the pollution problem**,** industrial wastes can be used in concrete composite materials. This is especially beneficial in places prone to earth quack and lower temperature. In this study, five different types of waste fibres such as polyester waste, rubber waste, rock wool waste, glass fibre waste and coconut fibre waste were used as an additive in 0.5% 1%, and 1.5% by mass in concrete mix. Seismic performance related properties of the samples were examined through evaluation of compressive strength, flexural strength, impact strength**,** split tensile strength, and thermal conductivity. Results showed that, impact strength of the concrete significantly improved by the addition of fibre reinforcement in concrete. Split tensile strength and flexural strength were significantly reduced. Thermal conductivity was also influenced by addition of polymeric fibrous waste. Microscopic analysis was performed to examine the fractured surfaces. In order to get the optimum mix ratio, multi response optimization technique was used to determine the desired level of impact strength at an acceptable level of other properties. Rubber waste was found to be the most attractive option followed by coconut fibre waste for the seismic application of concrete. The significance and percentage contribution of each factor was obtained by *Analysis of variance* ANOVA (α = 0.05) and pie chart which showed that Factor A (waste fibre type) is the main contributor. Confirmatory test was done on optimized waste material and their percentage. *The order preference similarity to ideal solution* (TOPSIS) technique was used for developed samples to obtain solution (sample) which is closest to ideal as per given weightage and preference for the decision making. The confirmatory test gives satisfactory results with error of 6.68%. Cost of reference sample and waste rubber reinforced concrete sample was estimated, which showed that 8% higher volume was achieved with waste fibre reinforced concrete at approximately same cost as pure concrete. Concrete reinforced with recycled fibre content is potentially beneficial in terms of minimizing resource depletion and waste. The addition of polymeric fibre waste in concrete composite not only improves seismic performance related properties but also reduces the environmental pollution from waste material which has no other end use.

## Introduction

Environmental concerns and energy efficiency are the two major issues of the present era. Concrete is the 2nd most widely used material in the world^1^. According to a report of World business council, 3.8 tons of concrete is being used per person annually^2^. The use of cement in large quantity has serious effects on environment because production of concrete material causes emission of carbon dioxide (CO_2_) and cement has a high level of toxicity, which is harmful to human life. Manufacturing of cement cause 7% of total manmade global CO_2_ emission all over the world^3^. Due to non-eco-friendly nature of cement, researchers are investigating alternative materials that can be sustainable. There are many attempts to overcome this issue. One of the options is to completely replace concrete component by other materials, but complete replacement is not possible because concrete has undisputable advantages. Partial replacement of concrete component with other environment friendly materials is a practical solution to this problem. Concrete has other drawbacks too like cracking, spilling, and brittleness besides being non-eco-friendly. Pure concrete is weak in tensile strength, although it has high compression strength. In order to prevent the environmental disaster arising due to large scale use of cement, concrete industries have been interested into reducing CO_2_ discharge, recycling resources and the development of alternative durable materials. Fibre reinforced concrete (FRC) is one of the cheapest and durable methods for modern construction industry as waste fibres are used for partial replacement of cement to provide required seismic performance while minimizing cost. Using fibres/waste leads to reduction in consumption of cement which helps in the construction of affordable housing. Many tons of waste are produced by the industries which has no end use. The natural decomposition of these waste materials takes very long time, and this type of waste remains as landfill. Such industrial waste includes polyester, rubber, cotton, plastic, rock wool, glass fibres, nylon, etc. Decomposition of polyester takes almost 20–200 years, for rubber takes 50–80 years, rock wool requires 1–5 years, and glass fibres take 4000–5000 years. This type of industrial waste can be used as reinforcement in construction industries. The use of waste in construction is a good step towards eco-friendly construction. Many researchers are working on using this type of waste in construction industry^4–6^.

Earthquakes occur around the world leading to disasters. The building structures which are un-reinforced or reinforced with steel collapse when subjected to seismic load due to insufficient ductility and strength. Collapse of the building structures cause injuries, loss of life and economical loss. The building structure should be strong enough to withstand the earthquake as much as possible. Therefore, it is necessary to use those reinforcement materials which increase strength, ductility and lateral strain of the building during seismic load. Researchers are trying to find the construction material or reinforcement which can withstand the seismic load^7–9^. Fibre or polymeric waste reinforced concrete is an emerging technology to save the building structure during seismic load or earthquake related disaster as they have enough strength and ductility to withstand the seismic load. The fibres are added as a partial replacement of cement, steel, and coarse aggregates. Some researchers used steel wires to reinforce the concrete structures which increase durability and reduce surface crack formation, but this method has not improved the seismic load bearing properties of reinforced concrete structures significantly^10,11^. Another study found that using steel bars as reinforcement in concrete can increase the load bearing capacity of column but not enough to withstand the seismic load^12^. Also, one of the drawbacks of using steel wire is that it is prone to rusting besides increasing structural weight and causing balling effect. The several types of fibres used as reinforcement in FRC are carbon fibre, glass fibre, aramid fibre, jute fibre, and polypropylene fibre etc. The use of high-performance fibres in concrete increases the compression strength, strain hardening response and flexure strength of the concrete buildings. The seismic capacity of building increases when high performance fibres are used as reinforcement in concrete^13^. Carbon fibre sheets are mostly used for earthquake resistant buildings. The building structures made of carbon fibres are very expensive. Researchers studied different percentages of jute fibre and different type of soils in concrete to make earthquake resistance buildings. They concluded that by changing the type of soil and addition of jute fibre in concrete increases the ductility of the concrete. Other researchers used jute and sisal fibres in concrete buildings for earthquake resistance and compared the strength with carbon fibre reinforced concrete. The concrete columns for buildings with relatively lower heights, with jute and sisal fibre achieved strength equal to carbon fibre sheet while incurring 35% lower cost^14,15^.

Some studies found that addition of 1% waste rubber in concrete helps to increase the ductility of the concrete which increases the earthquake resistance of the buildings^16–19^. Other studies found that different percentage of glass fibres as reinforcement in concrete improves the overall performance of the concrete such as mechanical resistance and fire resistance property^20–23^. Some researchers used different percentages of recycled and waste rock wool in concrete to increase the thermal resistance of the buildings^24,25^. Some studies found that lower percentage of coconut fibre waste in concrete cam improve the mechanical strength of and reduce the weight which has a positive effect during seismic loads. The addition of coconut fibre in concrete has positive effect on compression strength and negative effect on flexural strength^26–29^. Different types of industrial and domestic wastes such as PET bottles, face masks, and plastic straws etc., were used as reinforcement which increase the ductility of the concrete and reduce the compression strength and load bearing capacity of the concrete^30–33^.

Although different types of waste materials were used to produce lightweight concrete and building materials but it is still a challenge to produce lightweight building with greater load bearing capacity at an affordable cost. There are few or no research reported on the combined study of improvement in mechanical properties and thermal insulation of concrete material using industrial waste.

The overall aim of this study is to produce higher load bearing, low cost, and energy efficient sustainable building materials by addition of industrail fibrous waste in concrete as reinforcement. The obtained results are also compared with plain concrete (PC) samples and comprehensive study of improvement in thermal insulation and mechanical strength are presented. An important issue in materials engineering is to find optimum sample of FRC with desired quality of the concrete for particular applications. By the addition of waste material/ fibres some properties are improved while some others showed negative effect. For example, by addition of glass fibres in concrete, the mechanical strength is reduced but thermal insulation improves^34^. To get the desired properties in concrete different multi response optimization method, were used. Researchers used second order mathematical model for optimum design of concrete containing steel fibre as reinforcement^35^. Others used response surface method to countify the binder content in concrete reinforced with ultra-high-performance reinforced concrete^36^. Some of the decision-making problems which have multiple responses cannot be optimized at once because multiple objectives conflict with each other. Some of the responses have positive and some have negative effects on the desired properties. For this purpose, multi response optimization methods are used. Researchers used Technique for Order of Preference by Similarity to Ideal Solution (TOPSIS) to get the desired percentage of reinforced polymer blend in concrete^37^. Some other researcher used Analytical Hierarchy Process (AHP) to define the rank of the decision matrix and to calculate criteria weight of the matrix^38,39^. *The order preference similarity to ideal solution* (TOPSIS) technique is used for developed samples in order to obtain solution (sample) which is closest to ideal as per given weightage and preference for the decision making. In this work TOPSIS method was used to get the optimum material and percentage for reinforcement which can be used further for seismic applications. This research addresses the important environmental issues in terms of CO_2_ emission due to manufacturing of cement, energy consumption and waste disposal^40^. The significance of the research is that different types of industrial waste materials are used as partial replacement of cement which reduced the consumption of cement. This approach reduces CO_2_ emission by reducing the production of cement. It optimizes the handling of waste which would otherwise remain as landfill for several years. A sustainable solution for low-cost earthquake resistance building was achieved.

## Materials and methods

The concrete samples were prepared with ordinary Portland cement, sand with fineness modulus of 2.42, water: cement ratio 0.55, coarse aggregates of 4 mm size and five types of fibrous waste materials in 3 different percentages each. These waste fibrous materials were both from natural and synthetic origin. Coconut and rock wool are from natural origin while polyester, glass and rubber are from synthetic origin. The reinforcement fibres were used with 0.5%, 1% and 1.5% of the weight of cement.

### Materials

**Figure 1:**
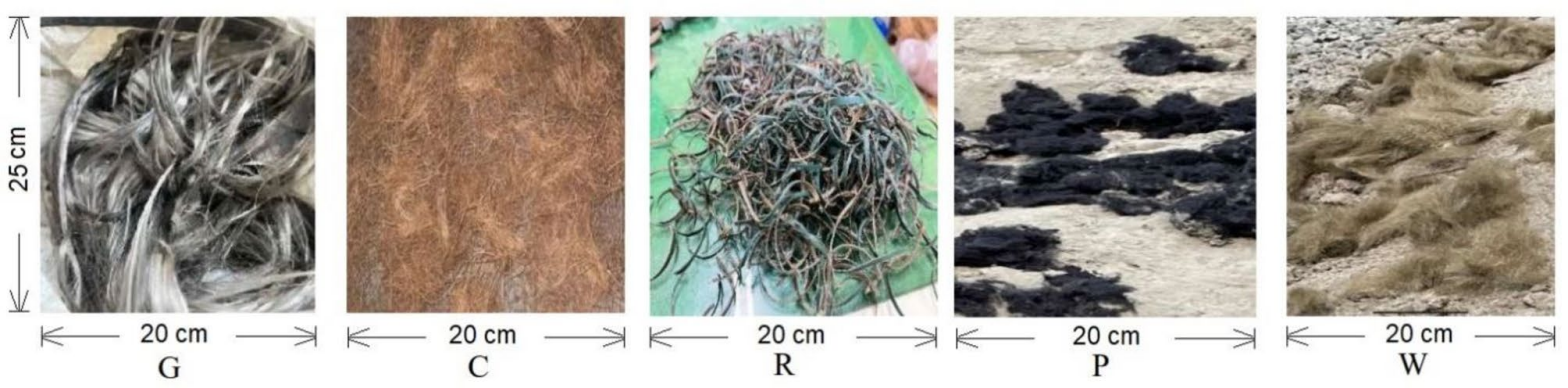
(G) Glass waste, (C) Coconut waste, (R) Rubber waste, (P) Polyester waste, (w) Rock wool waste.

Coconut fibres were taken from the waste of matt or rope industry. Polyester wastes were collected which are produced during the shearing process of woven fabrics. Waste glass fibres were collected from reverse osmosis (RO) water plant filters. Waste rubber, which is used in textile twister machinery was collected from rags market. Waste rock wool was taken from useless boilers. All the materials are waste which would otherwise remain as land fill and have no other end use. All the waste fibres were cut into length of 70 mm as shown in Fig. 1.

The strength of the fibres was evaluated by using Universal Testing Machine, as per standard ISO 5079. The properties of the fibres are given in Table 1.

**Table 1:** Details of reinforcement.

Sr.#	Industrial waste	ID	Strength (N)	Diameter (mm) 28% magnification	Average length of fibre (mm)	Aspect ratio (L/D)
1	Coconut	C	5.69	51.34	1000	19.48
2	Polyester	P	0.88	96.02	1000	10.41
3	Rubber	R	432.00	154.34	1000	6.48
4	Glass	G	30.33	3.70	1000	270.00
5	Rock wool	W	0.02	2.37	1000	421.94

### Methods

**Figure 2:**
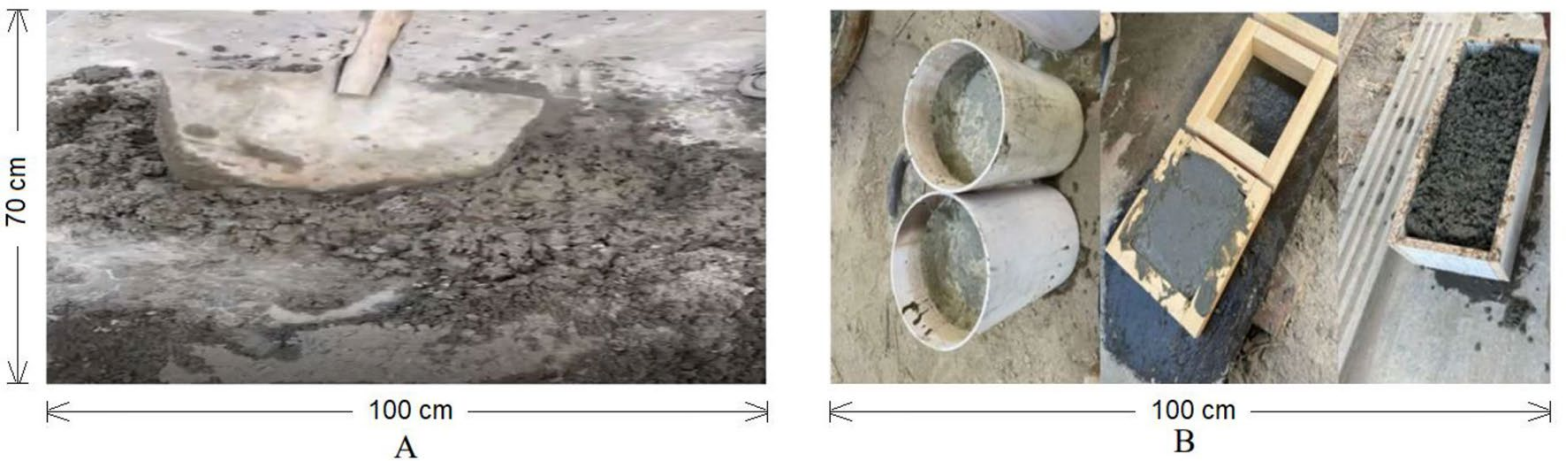
(**a,b**) Mixing of concrete (**c–e**) Moulds.

To prepare the samples of pure concrete (reference sample), the mix ratio of 1:2:3 (cement: sand: coarse aggregate), with water 55% by mass was used. Five different industrial wastes i.e., coconut fibre waste, polyester fibre waste, glass fibre waste, rock wool fibre waste and waste rubber were added as reinforcement in mixture to make FRC. Three different ratios i.e., 0.5%, 1% and 1.5% of each waste material were used on weight of the cement to make the concrete samples. All the materials were mixed as shown in Fig. 2. Then the mixture was cast into different moulds for different tests according to standards as shown in Fig. 2. Total 48 samples were casted for 28 days for each test. Three replications of each sample were prepared to ensure the repeatability of the tests conducted. Design of experiment and sample details are shown in Tables 2 and 3.

**Table 2:** Design of experiments.

Sr #	Factors	ID	Levels				
			1	2	3	4	5
1	Reinforcement/industrial waste	A	Waste coconut fibre	Waste polyester fibre	Waste rubber fibre	Waste Glass fibre	Waste rock wool fibre
2	Fibre percentage (%)	B	0.5%	1%	1.5%

**Table 3:** Factors and their level.

Sample no	Sample ID	A	B
1	PC	Plain concrete	Nil
2	C1	Coconut fibre	0.5
3	C2	Coconut fibre	1
4	C3	Coconut fibre	1.5
5	R1	Rubber fibre	0.5
6	R2	Rubber fibre	1
7	R3	Rubber fibre	1.5
8	P1	Polyester fibre	0.5
9	P2	Polyester fibre	1
10	P3	Polyester fibre	1.5
11	G1	Glass fibre	0.5
12	G2	Glass fibre	1
13	G3	Glass fibre	1.5
14	W1	Rock wool Fibre	0.5
15	W2	Rock wool fibre	1
16	W3	Rock wool fibre	1.5

### Testing

#### Mechanical testing

Concrete during its service life goes through different types of loading, which produce cracks on the surface as well as the inner portion. Mechanical loadings are of different types and crack formation can be controlled by improving the required properties. Cracks produced on the surface of rigid pavement are due to lower flexural strength. Similarly concrete spalling phenomenon can be controlled by improving the tensile strength. The resistance against impact loading can be improved by enhancing energy absorption property of concrete. Impact property is very important with respect to blasting, collision of vehicle especially on bridges and during earthquake. All the samples were tested with a replica of 3 for all three different fibre percentages for each type of waste fibre as reinforcement in concrete.

#### Compressive strength test

**Figure 3:**
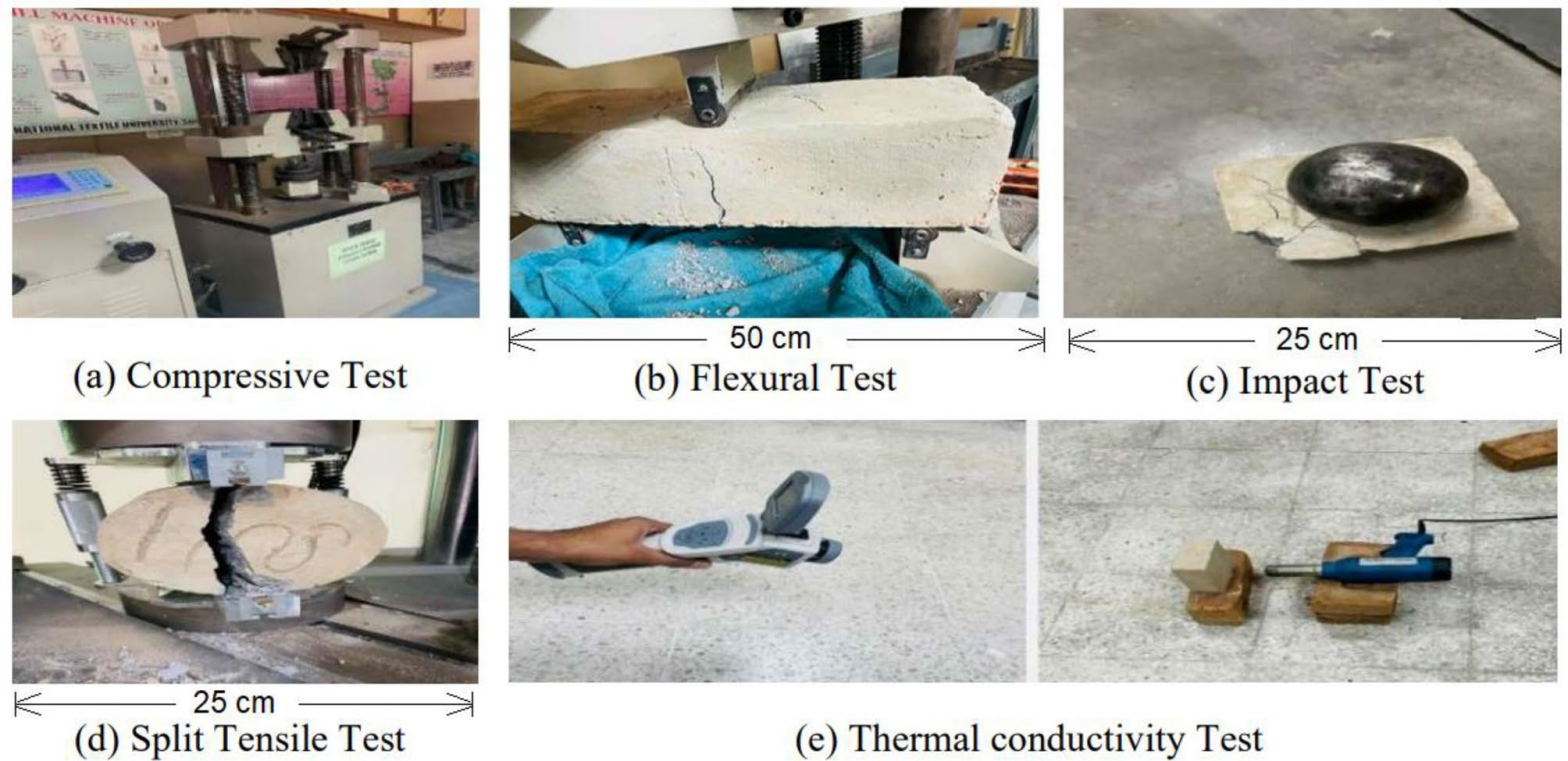
Testing Instruments.

Compressive strength of the concrete samples of dimension 100 mm × 100 mm × 100 mm were measured by using Digital-Display Hydraulic Universal Testing Machine (UTM) (Model: Beijing Sino found WES-100) as shown in Fig. 3. For compression test, a total 48 samples were prepared with 3 replicates for each sample. Each specimen was placed vertically between the jaws so that it acts as a prototype of compression member or a column. In this test the concrete samples were placed between two jaws one of which was moveable and other was stationary. When the load is applied through jaws, the point at which the failure of concrete sample occurs shows as a digital reading, which is the total compression strength of the concrete sample.

#### Flexural strength

The flexural/bending test of the concrete samples was carried out with dimensions,150 mm width × 150 mm depth × span of 500 mm., by using Digital-Display Hydraulic Universal Testing Machine (Model: Beijing Sino found WES-100). Testing standards for flexural strength ASTM C78/C78M-21 was used^41^. For measuring the bending strength of the concrete total 48 sample were prepared with 3 replicates. For measuring the bending strength of the concrete, the samples were placed on two rods with equal distance from edges i.e. 75 mm and one rod was on opposite side at centre of the samples as shown in Fig. 3.

#### Impact energy

Due to unavailability of a specific standard to determine the impact properties of the concrete by drop weight test, the method as adopted by other researchers was used^42^. Sample dimension taken were (L × W × T) 125 mm × 100 mm × 50 mm. In this method a steel ball of weight 1.8 kg was taken and dropped from the fixed height of 889 mm on the test specimen. The test continued until the failure in sample occurs as shown in Fig. 3c. Total 48 samples were prepared for impact test. The impact energy of samples was calculated by multiplying mass of the ball, height of the ball drop, acceleration due to gravity and number of blows needed for the sample before fracture. The impact energy was calculated by using Eq. (1)^43^.

1 $$ E\, = \,N \times m \times g \times h, $$

where, *E* is total impact energy at failure of the sample; *N* is the number of blows at failure of the sample; *m* is the mass of the ball which is 1.8 kg; *h* is the weight of the ball drop, which is 889 mm; and *g* is the acceleration due to gravity i.e. 9.81 m/s^2^.

#### Split tensile strength test

Hydraulic universal testing machine (IBMU4 series) was used to perform the split tensile strength test of the lightweight concrete samples as shown in 3(d). Cylindrical specimens were laid down between the testing plates for performing splitting tensile test. For the testing of sample, the standard size of the specimen (H × D) 250 mm height and 150 mm diameter was used.

#### Thermal conductivity/infrared thermography (IRT)

A building/construction acts as external envelop/barrier that separates the outer and inner environments, and it protects the habitants from external environment. Reducing the cost of heating/cooling of the building directly contributes to energy efficiency and sustainability.

Due to the increasing use of infrared thermography in the building sector, for detection of cracks etc., a new method was adopted for measuring thermal conductivity^44^. Thermal properties of the samples were determined by using Thermal imaging camera CANTRONIC System Inc. (Canada) and the specimens were heated by heat gun as shown in Fig. 3e.

Only, FRC with 1% of waste materials and the reference sample were measured by using Thermal imaging camera. Cubic samples with dimensions height/thickness 50 mm, width 100 mm and length 125 mm were prepared. First, the samples were placed properly for 4 min to make a steady flow of heat. The power of the apparatus was set at 10 watts and the temperatures of both side of the sample were recorded. Then after 5 min, the temperature and thermal images of both sides of the samples were taken again. Temperatures from thermograms were analysed using thermal imaging software, (IR Camera Report software). Then, thermal conductivity of the samples was calculated using Eq. (2)^45^. Heat transfer, area and thickness were constant, while T1 and T2 are variables.

2 $$k=\frac{Q \times t}{A \times \mathrm{\Delta T}},$$

where: *K* = Thermal conductivity, W/mK, *Q* = Power supplied during the test, W, *A* = Cross-sectional area, m^2^, *t* = thickness, m, ΔT = (T1 − T2) = Temperature difference between first point in hot region and last point in cold region, K.

### Microanalysis

The fractured surfaces of broken specimens were examined carefully after mechanical testing. Purpose of this examination is to elaborate the failure mechanism and check the bonding of fibres with concrete. For this purpose, microscopic images of the samples were taken using OPTIKA microscope (BX53, Olympus) with the magnification power of 2.5 ×. In this examination pull out, bridging effect and fibre breakage was investigated.

### Statistical analysis

Design of experiment was done by Taguchi method as it is one of the most common methods in scientific research. But its limitation is that it can deal with only single response optimization. So, it cannot be used directly for multi-response optimization. Optimization techniques are the best solutions for such research. Different types of modelling and simulation techniques can be used depending upon the experimental results to find the optimum mix ratio for concrete. Mostly for complex linear decision matrix, FUZZY logic technique can be used. FUZZY logic method is normally used to find the unknown variable. Like multi criteria decision making (MCDM), TOPSIS method can be used to find the ideal solution. In this work AHP-TOPSIS Method was used to convert multi responses results into single response. TOPSIS (The order preference similarity to ideal solution) technique was further developed by researchers^46–49^. AHP was used to find the weightage of the different attributes which has a scale of 1–9 showing equal importance to all the attributes. According to the desired seismic performance for earthquake resistance, the building should be strong in compression and should exhibit enough split, and flexural strength. Compression strength is highly important in this analysis and given a number of 5. Flexural strength and impact strength has importance between strong and moderate and given number 4. Split tensile strength was given number 3 as it has moderate importance in case of earthquake resistance. AHP used to find the weightage of the different attributes has a scale of 1–9 which shows equal importance to the attributes.

TOPSIS consists of the following steps.

1. TOPSIS method begins with decision matrix which contains different attributes. The lowest, highest and nominal values of them are considered. In this experimental study, higher the factor, is considered as better. The order of preference for this technique is impact, tensile, flexural and compression.
  3 $$ {\text{D}}\,{ = }\,{\text{Xij}} $$
2. In the 2nd step the attributes are normalized by dividing each value of attribute by some of square root of respective attribute using Eq. (4).
  4 $$\mathrm{rij} = \frac{\mathrm{Xij}}{{\surd {\varvec{\Sigma}}\mathrm{ij}}^{2}}$$
3. In this step the weights of the different attributes are determined according to preference of different attributes with respect to objective. Preference scale can be used as.
  5 $$\mathrm{Wi }= \frac{(\mathrm{aij})}{\surd {\varvec{\Sigma}}\left(\mathbf{a}\mathrm{ij}\right)}$$
4. The calculated weights of different attributes are normalized by multiplying the weights with normalized values of each attribute.
  6 $$ {\text{V}}_{{{\text{ij}}}} {\text{ = W}}_{{\text{i}}} \times {\text{ r}}_{{{\text{ij}}}} $$
5. Positive ideal solutions (v +) and negative-ideal solutions (v −) are calculated by following method,
  7 $$ {\text{A}}^{ + } \, = \,\left\{ {\left( {\max \,{\text{V}}_{{{\text{ij}}}} \left| {{\text{j}}\,{{\EUR}} \,\,\mathop {\text{J}}\limits^{.} } \right.} \right),\,\left( {\min \,{\text{V}}_{{{\text{ij}}}} \,\left| {{\text{j}}\,{{\EUR}}\,\,\mathop {\text{J}}\limits^{.} } \right|\,{\text{i}}\,{ = }\,{1,}\,{2,}\, \ldots {\text{m}}} \right)} \right\}\, = \,\left\{ {{\text{y}}_{1} ,\,{\text{y}}_{2} ,\, \ldots {\text{Y}}_{{\text{j}}} ,\, \ldots {\text{y}}_{{\text{n}}} } \right. $$
  8 $$ {\text{A}}^{{^{ - } }} \, = \,\left\{ {\left( {\min \,{\text{V}}_{{{\text{ij}}}} \,\left| {{\text{j}}\,{{\EUR}} \,\mathop {\text{J}}\limits^{.} } \right.} \right),\,\left( {\max \,{\text{V}}_{{{\text{ij}}}} \,\left| {{\text{j}}\,{{\EUR}} \,\mathop {\text{J}}\limits^{.} } \right|\,\,{\text{i}}\,{ = }\,{1,}\,{2,}\,{3,}\, \ldots {\text{m}}} \right)} \right\} $$

*J* is associated with beneficial while *J*^*ˈ*^ is associated with non-beneficial factor.

1. In this step distance can be measured by separating each alternative from the ideal solution.
  9 $$ {\text{S}}_{{\text{i}}}^{ + } \, = \,\surd \Sigma \left( {{\text{y}}_{{{\text{ij}}}} - {\text{y}}_{{\text{j}}} } \right)^{{2}} {\text{ i}} = 1,\,2, \ldots {\text{m}} $$
  10 $$ {\text{S}}_{{\text{i}}}^{ - } \, = \,\surd \Sigma \left( {{\text{y}}_{{{\text{ij}}}} - {\text{y}}_{{\text{j}}} } \right)^{{2}} \, {\text{i}} = {1},\,{2}, \ldots .{\text{m}} $$
2. In the final step, closeness coefficient can be calculated by using Eq. (11).
  11 $${\mathrm{Ci}}^{+} = \frac{\mathrm{Si}-}{Si+Si-}$$

Analysis of Variance (ANOVA) was done by using Minitab software (www.minitab.com) to determine whether the factors were significant or not. For this purpose, the p-values were examined. Percentage contribution of each factor was also shown on pie chart.

## Results and discussion

The results of all the tests performed are shown in Table 4.

**Table 4:** Samples and their response.

Symbol	Compressive strength (kN)	Flexural strength (kN)	Impact energy (J)	Split tensile strength (kN)	Thermal conductivity (W/mK)
PC	105.79	25.59	47.04	132.34	910
C1	67.95	11.81	62.73	48.08	–
C2	90.21	17.02	78.41	95.49	512
C3	62.16	12.11	78.41	51.46	–
R1	105.93	26.11	62.73	102.52	–
R2	106.55	19.01	101.93	86.42	956.65
R3	107.87	15.93	94.09	75.65	–
P1	59.91	12.88	54.89	52.71	–
P2	80.77	13.31	70.57	68.24	577.62
P3	67.81	9.13	70.57	60.80	–
G1	53.27	15.44	62.73	67.31	–
G2	84.32	17.35	62.73	85.32	540.99
G3	89.24	22.96	60.57	90.21	–
W1	88.14	16.31	47.05	71.72	–
W2	108.66	19.02	78.41	84.17	633.66
W3	93.12	16.39	70.57	57.49	–

### Compressive strength

**Figure 4:**
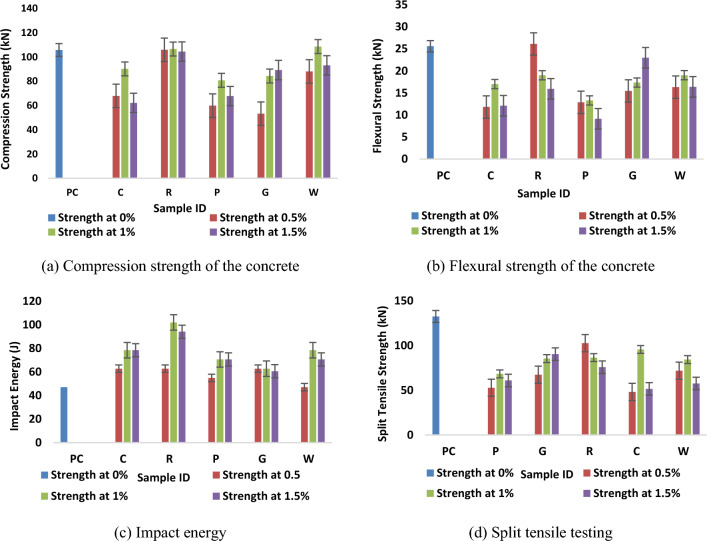
Mechanical performance of fibre reinforced concrete vis-à-vis pure concrete (**a**) Compression, (**b**) Flexural, (**c**) Impact and (**d**) Split tensile properties.

The strength of the concrete depends upon the strength of the fibre, aspect ratio, orientation of fibre and bonding between fibre and matrix. Instead of spalling like PC, diagonal and shear cracks were appeared on samples of FRC under compressive loading. The compressive strength of the concrete with different waste fibres and different percentages of reinforcement was tested. From the Table 4 and Fig. 4a, the results show that with 0.5% addition of reinforcement, there is decrease in compressive strength for all the FRC, but there is no significant change in compressive strength of rubber waste reinforced concrete. When the percentage of reinforcement increased to 1%, then the samples show slight increase in compressive strength as compared to 0.5% loading of fibre waste. Only rubber waste and rock wool reinforced concrete show significant increase in compressive strength, much greater than PC while others show decreasing trend in compressive strength. The Rubber waste reinforced concrete showed maximum compressive strength at all levels of reinforcement and Rock wool-based sample showed increase only with 1% fibre loading. At 1% loading of reinforcement, the increase in compressive strength is 1% for rubber waste and 20% for rock wool. Interfacial transition zone between the cement paste and the rubber waste filled the voids, thus leading to increase in compressive strength. Rubber alone does not have a perfect bonding with the matrix of cement but in case of rubber waste (blend of nylon and styrene butadiene rubber), nylon provided perfect bonding and styrene butadiene provided enough strength to the concrete as seen in Fig. 4. By adding rubber, there is change in the failure mode of concrete from brittleness to ductility. Rock wool also has good bonding with the matrix of the concrete; and thus, results show improvement in compressive strength. Polyester has poorest bonding strength with the matrix of concrete and therefore, shows significant reduction in the compressive strength as compared to other fibres. Coconut fibre reinforcement shows decrease in compressive strength because the strength of the fibre is reduced in the concrete due to absorption of water. In case of coconut fibre reinforcement, the bond between fibre and matrix is not perfectly formed due to presence of dust on the porous surface of fibre^50–52^.

### Flexural strength

The flexural strength of the concrete sometimes reduces by the addition of the reinforcement as shown in Table 4. At 0.5% waste fibre loading, all the samples show decreasing trend except sample R1 as shown in Fig. 4b. With increasing fibre percentage to 1%, some samples show improvement in flexural strength. Further addition of fibre to 1.5% causes reduction in flexural strength in all the samples. The reason for improvement in flexural strength in sample R1 is that the reinforcement in this case is the blend of nylon and styrene butadiene rubber (SBR). Nylon formed perfect bond with the matrix of cement while SBR provided strength and some ductility. Bridging effect of reinforcement areas with surrounding matrix is achieved. The sample takes more load and crack propagation is delayed. The samples which showed lower flexural strength may be due to accumulation of reinforcement at certain places in concrete. This tends to reduce flexural strength of the concrete at certain points^53^. The flexural strength of the concrete reduced at higher percentage of reinforcement because there is chance of interlocking of the reinforcement and more air is trapped inside the concrete sample which results in reduction of flexural strength^54,55^. Further the reduction is a result of lower flexural rigidity of the fibres as compared to concrete as brittle material.

### Impact energy

Impact energy of the concrete samples increased by the addition of fibrous waste. At 0.5% reinforcement in concrete, the improvement is observed in all samples as shown in Table 4 and Fig. 4c. PC is a brittle material, while addition of reinforcement helps in ductility. The increase in impact energy is 25% for rubber, glass and coconut fibre reinforcement. The improvement in impact energy in case of polyester reinforcement was 13%. There is no significant improvement in impact energy of rock wool reinforced concrete. At 0.5% the improvement in impact energy was attributed to macro fibres which is used in concrete. This type of macro fibres resists the initiation of cracks under stresses and absorb more energy than non-fibrous concrete material^55^. When the percentage of reinforcement in concrete increased to 1% the rubber waste shows maximum increase in impact energy about 54%. Coconut fibre, rock wool, polyester and glass show 40%, 40%, 33% and 25% improvement in impact energy respectively with respect to PC. Rubber reinforcement in concrete might be bonded with the surrounding matrix and reduce voids. This results in stronger structures which shows improved strength under impact force. The increased fraction of reinforcement effectively reduces the propagation of macro cracks in concrete^53^. These types of fibre reinforced concrete which exhibit enough ductility can be used in anti-seismic applications^49–53^. Further addition of reinforcement tends to reduce the impact energy in all the samples that may be due to chance of interlocking of the reinforcement which results in reduction of overall energy absorption.

### Split tensile strength

The tensile strength of the concrete is not accurately measured by split tensile testing method because of mixed stress field and different fibre orientations. Split tensile strength is useful because the failure pattern of the concrete sample is defined as brittle or ductile. By adding different reinforcement in concrete, the split tensile strength of the concrete reduced significantly, but the ductility of the samples is improved. The samples reinforced especially with rubber and coconut fibres split in to two pieces by first crack propagation. Due to bridging effect of rubber and coconut reinforcement, there is better load-transfer to the fibres which tend to show an improved ductility. Similar results for seismic performance were achieved by other researchers^53–55^. Theses fibres in concrete reduced the post crack failure in concrete and increase the ductility of the concrete. It may also be predicted that the FRC can sustain a little longer against split tensile forces as compared with PC because fibre reinforcements present in concrete act as crack arrestors and resist the crack propagation. The bar charts of split tensile strength of the concrete samples are shown in Fig. 4d. Since split tensile test is similar to a low width bending test, the results are very much similar to bending test.

### Thermal conductivity

**Figure 5:**
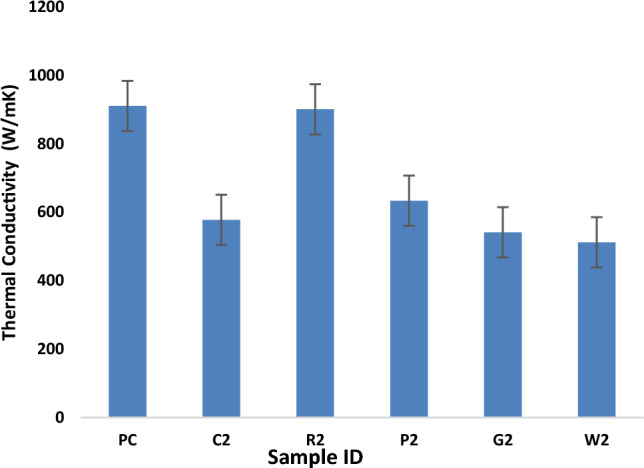
Thermal Conductivity.

**Figure 6:**
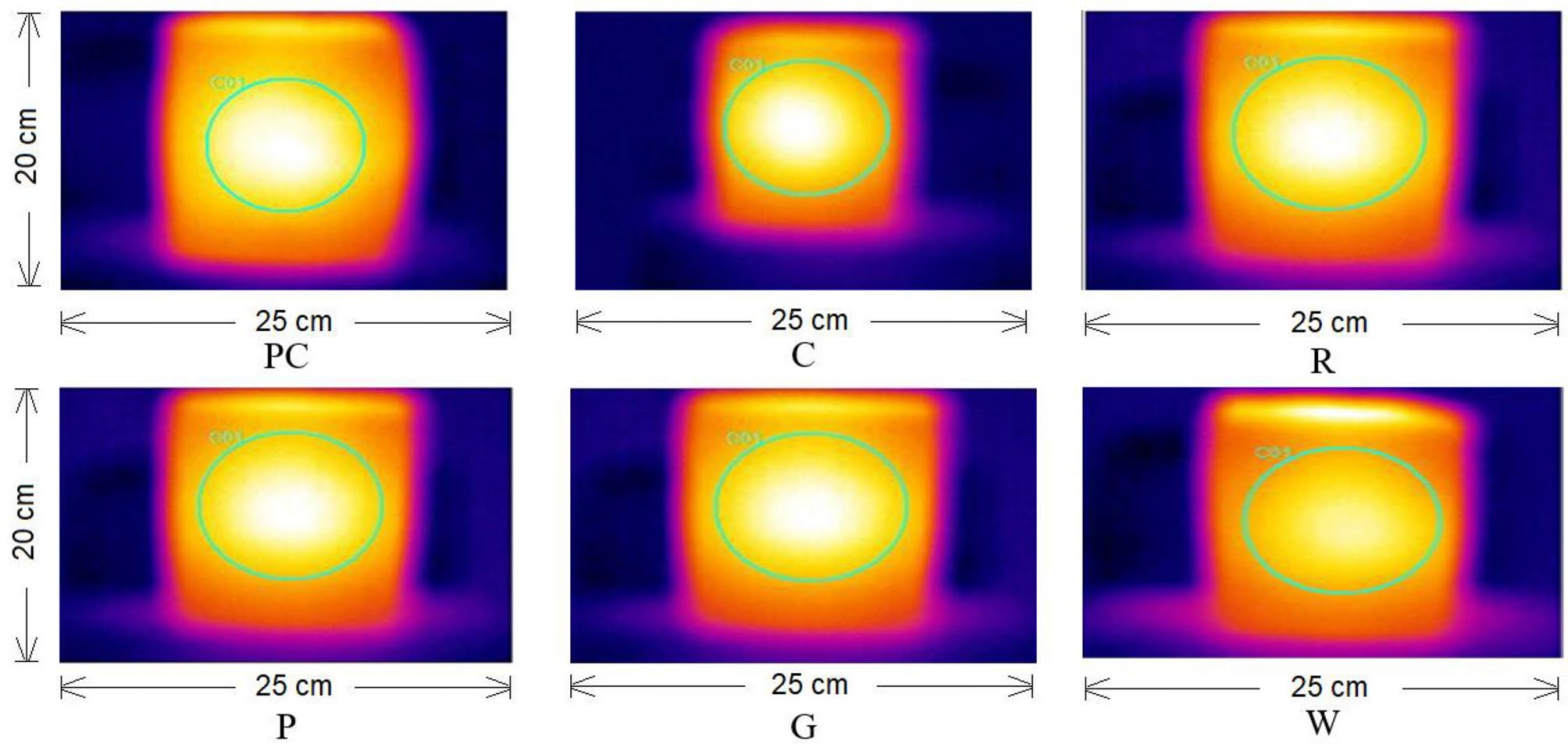
Illustrations of thermal images of samples.

Thermal conductivity is an important parameter to access the material property, if it is a good insulator or not. The thermal conductivity values of the 1% fibre reinforced concrete samples were evaluated to detect the thermal behaviour. The thermal conductivity of all the samples at 1% reinforcement dosage are given in 4. The trend of thermal conductivity is shown in Fig. 5. The results show that the rock wool fibre reinforced concrete shows lowest thermal conductivity with 43% decrease from reference sample. Glass, polyester, and coconut fibre reinforced concrete samples show 41%, 37% and 31% decrease in thermal conductivity respectively when compared with reference sample PC. Therefore, rock wool and waste glass fibre reinforced samples show higher thermal insulation property. The thermal conductivity of the concrete is significantly reduced by fibrous content due to the porosity of the reinforcement. The concrete containing rock wool and waste glass fibre as reinforcement can be used in thermal insulation of buildings. Thermal images of all the samples after 5-min exposure to heat source are shown in Fig. 6.

Thermal imaging quantifies the temperature of the other side of heated surface of samples. The samples having higher thermal resistance have shown lowest heat dissipation/ heat loss as shown in the results of thermal imaging in Fig. 6. Rock wool, glass, coconut and polyester fibre reinforced samples show higher thermal resistance as depicted in the Figure. The lighter region shows lower heat transfer. PC sample without any reinforcement shows the highest heat transfer indicated by maximum brightness.

### Microscopic images of cracked surfaces

**Figure 7:**
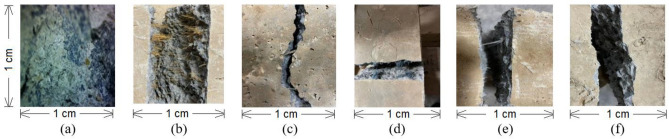
Microscopic images of cracked samples (**a**) Pure concrete (**b**) Coconut fibre (**c**) Rubber waste (**d**) Rock wool waste (**e**) Polyester waste (**f**) Glass waste.

Microscopic images of the broken waste fibres reinforced concrete samples were taken to check the bonding pattern of fibre with the surrounding concrete matrix. After the application of loads during mechanical testing, the samples broke into pieces. The images of fractured surfaces are shown in Fig. 7. The attachment of small fragments and hanged particles indicates the bridging effect of fibres in the concrete. Due to the bridging effect of reinforcing fibres, the concrete can avoid sudden failure and show higher post crack energy as compared to PC. The images show that strong bonding is present between fibrous waste and cement. The pull out of waste fibre can be observed in some cases in the images. Similar observation was made for coconut fibre. However, Polyester waste has minimum bonding with concrete because the fibres are mostly hydrophobic and have lower affinity towards the concrete. It is concluded after performing experiments that the reinforcement of fibrous waste in concrete helps to resist the initiation and progression of cracks.

### TOPSIS analysis

All the experimental data from Table 4 referring to mechanical performance are used to form a matrix in which rows represent alternatives and columns represent attributes of decision matrix. The table is analysed by the mentioned procedure of TOPSIS based Taguchi method. In the first step, the matrix is normalized by using Eq. (4). The matrix is normalized by dividing each attribute value by sum of square root of all the attribute values in that category. For example, the value of compression strength of PC is 105.79 which is divided by sum of square root of all the value of compression strength that is 350.56. This gives the normalized value of 0.30. All the normalized values are given in Table 5.

**Table 5:** Normalized value of Decision matrix/ Normalized Signal to noise ratio.

Sample ID	Compression strength (kN)	Flexural strength (kN)	Impact energy (J)	Split tensile strength (kN)
PC	0.30	0.36	0.16	0.41
C1	0.19	0.17	0.22	0.15
C2	0.26	0.24	0.27	0.30
C3	0.18	0.17	0.27	0.16
R1	0.30	0.37	0.22	0.32
R2	0.30	0.27	0.35	0.27
R3	0.30	0.23	0.33	0.24
P1	0.17	0.18	0.19	0.16
P2	0.23	0.19	0.25	0.21
P3	0.19	0.13	0.25	0.19
G1	0.15	0.22	0.22	0.21
G2	0.24	0.25	0.22	0.27
G3	0.25	0.33	0.22	0.28
W1	0.25	0.23	0.16	0.22
W2	0.31	0.27	0.27	0.26
W3	0.26	0.23	0.24	0.18

Weight of each decision matrix is found by AHP method. Weights are given according to importance of the property such as compression strength has strong importance with a given no 5, flexural strength and impact strength are given no 4 since they have importance between strong and moderate. The split tensile strength has moderate importance and is given no 3. The weight and weighted normalized values are calculated by using Eqs. (5) and (6). Normalized weight are given in Table 6.

**Table 6:** Weighted Normalized Matrix.

Sample ID	Compression strength (kN)	Flexural strength (kN)	Impact energy (J)	Split tensile strength (kN)
PC	0.02	0.05	0.08	0.11
C1	0.01	0.02	0.11	0.04
C2	0.02	0.03	0.14	0.08
C3	0.01	0.02	0.14	0.04
R1	0.02	0.05	0.11	0.08
R2	0.02	0.04	0.18	0.07
R3	0.02	0.03	0.17	0.06
P1	0.01	0.02	0.10	0.04
P2	0.02	0.03	0.13	0.05
P3	0.01	0.02	0.13	0.05
G1	0.01	0.03	0.11	0.05
G2	0.02	0.03	0.11	0.06
G3	0.02	0.04	0.11	0.07
W1	0.02	0.03	0.08	0.06
W2	0.02	0.04	0.14	0.07
W3	0.02	0.03	0.13	0.05

The positive and negative ideal solutions were calculated by Eqs. (7) and (8). The separation distance from positive and negative ideal solution can be calculated by using Eq. (9). In the final step, by using Eq. (10) the overall performance coefficient closest to ideal solution was calculated. Closeness coefficients are shown in Table 7.

**Table 7:** Closeness coefficient/Si + , Si − and Ci.

Sample ID	Sum of all rows of Negative ideal solutions (Si −)	Sum of all rows of Positive ideal solutions (Si +)	Closeness coefficient (Ci)	Rank of the attributes
PC	0.010	0.02	0.24	5
C1	0.000	0.02	0.04	14
C2	0.010	0.02	0.31	3
C3	0.003	0.02	0.20	8
R1	0.004	0.02	0.21	7
R2	0.010	0.01	0.80	1
R3	0.008	0.01	0.56	2
P1	0.000	0.02	0.01	16
P2	0.002	0.02	0.12	9
P3	0.002	0.01	0.11	11
G1	0.001	0.02	0.06	13
G2	0.002	0.02	0.11	12
G3	0.004	0.02	0.22	6
W1	0.001	0.02	0.02	15
W2	0.004	0.01	0.29	4
W3	0.002	0.02	0.12	10

### Analysis of variance

Analysis of variance (ANOVA) was carried out at 95% confidence level to check the significance of the type of fibrous waste and the percentage of the reinforcement in concrete. Analysis of variance is shown in Table 8. The p-value which is 0.008 indicates the significance of fibre waste type at 95% confidence level while the percentage of reinforcement has p-value 0.019 indicating significant effect on the performance of the concrete.

**Table 8:** Analysis of Variance.

Source	Degrees of freedom	Adj SS	Adj MS	F-value	P-value
Model	6	0.555	0.092	7.36	0.006
Linear	6	0.555	0.092	7.36	0.006
A	4	0.383	0.096	7.62	0.008
B	2	0.172	0.086	6.84	0.019
Error	8	0.100	0.013		
Total	14	0.655			

**Figure 8:**
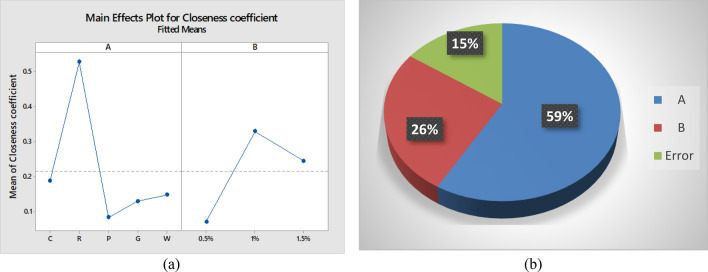
(**a**) Main effect plot and (**b**) Pie chart.

The main effect plot of closeness coefficient and pie chart of overall contribution of the factors are shown in Fig. 8. The main effect plot shows that the type of fibre greatly influences the performance of the concrete and waste rubber reinforced concrete proved to be the most effective reinforcement in concrete. Main effect plot shows that percentage of waste does not enhance the performance of the concrete significantly. The pie chat shows overall contribution of the factors. The type of waste fibre used as reinforcement has the highest contribution (59%) on the seismic performance of concrete.

### Confirmatory test

The closeness coefficient which has the highest value shows the optimum mix ratio of FRC. Confirmatory test was also done to verify the TOPSIS methodology. In confirmatory test the samples were prepared according to optimum mix ratio i.e., 1% rubber as reinforcement in concrete. Samples were prepared and tested to check the maximum error in the optimum mix ratio. The results of confirmatory test for all tests are shown in Table 9. The maximum error was calculated to be 6.681% which shows the satisfaction of the test results. Thus it was confirmed that 1% addition of rubber waste in concrete increases overall seismic performance of the concrete.

**Table 9:** Confirmatory test.

Responses	R^2^	Confirmatory test	Percentage (%) error
Compression strength (KN)	106.553	103.437	2.830
Flexural strength (KN)	19.013	19.200	0.973
Impact energy (J)	101.932	100.540	1.365
Split tensile strength (KN)	86.418	85.110	1.513
			Total error = 6.681%

### Cost estimation

The cost of PC and 1% rubber waste reinforced concrete was investigated to confirm that the replacement of cement with waste leads to reduce the cost of concrete significantly. First, the overall cost was calculated to make the PC samples which have total volume 11,250 cm^3^. The cement used for the samples costed 1 USD, sand costed 0.22 USD and coarse aggregates costed 1.10 USD. Total cost was 2.32 USD. By using waste rubber as 1% reinforcement in concrete, it occupied 8% more volume with the same cost incurred. Thus, FRC reduced the overall cost of the concrete which leads to low-cost/ affordable housing. The cost estimation is shown in Table 10.

**Table 10:** Cost Estimation.

Reinforcement	Cost of cement (USD)	Cost of sand (USD)	Cost of aggregates (USD)	Cost of waste or fibre (USD)	Total cost (USD)	Volume achieved (cm^3^)
Nil	1.00	0.22	1.10	0.00	2.32	11,250
Rubber waste	0.82	0.22	1.10	0.05	2.19	12,150

## Conclusions

Reuse of industrial waste is a worldwide phenomenon for a sustainable future. Burning/dumping waste causes damage to eco-system which leads to harmful impact to environment. In this study, different industrial fibrous wastes were used in concrete in different percentages. Overall, the concrete reinforced with waste fibrous materials not only improves seismic performance in terms of mechanical and thermal properties, but also reduces the waste handling problem. Compression strength of the concrete increased by the addition of rubber and rock wool waste at 1% and 2% loading respectively. Rubber waste at 0.5% addition shows 4% increase in flexural strength. With increasing percentage, the flexural strength of the concrete sample reduced. The impact energy of all the samples increased with addition of fibrous waste as reinforcement. The maximum increase in impact energy is with rubber waste at 1% loading. The split tensile strength of concrete was negatively affected by the addition of fibre waste. Thermal conductivity of the concrete was greatly reduced by the addition of fibre waste. Rock wool and coconut fibre reinforcement in concrete greatly reduced the thermal conductivity of the concrete making it an energy efficient construction material. From statistical analysis (TOPSIS) it was proved that sample R2 (with 1% rubber fibre) shows overall most favourable seismic performances. Addition of 1% rubber in concrete increases the compression strength, flexural strength, and impact strength of the concrete. So, sample R2 is recommended to be used for low-cost, earthquake resistance/seismic building. Hence, industrial waste can be utilized in a useful way as a construction material for a sustainable future. Also, by using waste fibre reinforcement, overall cost of construction will also decrease which will lead to low-cost housing with better seismic capacity for earthquake prone areas.

Future research can be conducted to use other types of agricultural waste and consumer waste in building construction. The performance can be evaluated at extreme temperatures to ascertain the performance in arctic and tropical environments. Further the influence of environmental factors e.g., humidity, salinity etc. can be investigated for durability of concrete reinforced with waste fibres.

## Data Availability

All data generated or analysed during this study are included in this published article.
