# Author Correction: Optimization of seismic performance in waste fibre reinforced concrete by TOPSIS method

**DOI:** 10.1038/s41598-023-37043-x

**Published:** 2023-06-20

**Authors:** Husnain Ali, Hafsa Jamshaid, Rajesh Mishra, Vijay Chandan, Petr Jirku, Viktor Kolar, Miroslav Muller, Shabnam Nazari, Khan Shahzada

**Affiliations:** 1 National Textile University, Faisalabad, Pakistan; 2 Department of Material Science and Manufacturing Technology, Faculty of Engineering, Czech University of Life Sciences Prague, Kamycka 129, 165 00 Prague, Czech Republic; 3 Department of Sustainable Technologies, Faculty of Tropical Agriscience, Czech University of Life Sciences Prague, Kamycka 129, 165 00 Prague, Czech Republic; 4 Civil Engineering, University of Engineering and Technology, Peshawar, Pakistan

Correction to: *Scientific Reports* 10.1038/s41598-023-35495-9, published online 21 May 2023

The original version of this Article contained an error in the spelling of the author Husnain Ali which was incorrectly given as Husain Ali.

The original Article has been corrected.

